# Thomas Jones

**Published:** 1934

**Authors:** 


### THOMAS JONES,

*Captain and Quartermaster, R.A.M.C.(T.);*

*Steward, Southmead Hospital.*

THOMAS JONES began his career with the Guardians of the Parish of Lambeth in 1896, and was subsequently Master of Alresford Workhouse, Hants., from 1903 to 1905. Then, coming to Bristol, he was successively Storekeeper and Master's Clerk at Eastville Institution until he was promoted to be Steward at Southmead Infirmary in September, 1914, in which capacity he watched over the conversion of the Infirmary into a military hospital. In May, 1915, he was gazetted Lieutenant and Quartermaster, R.A.M.C.(T.), and was promoted to the rank of Captain in May, 1918. Those of our readers who worked with him as Quartermaster will admit that it would be difficult to over-praise Mr. Jones's efforts during the War. His administrative competence had been proved before, but he handled his new task with satisfaction to everyone concerned. In 1920 Southmead Infirmary was handed back to the Guardians, and Mr. Jones became Steward, a post which he continued to fill with energy and enthusiasm, despite in the later years failing health, up to the time of his death at the age of 62 on 3rd April, 1934, after a short illness.

He leaves a widow and two sons, to whom our sympathies are extended.

